# Immunotherapy – 2080. Fel d 1 derived peptide antigen desensitization results in a persistent treatment effect on symptoms of cat allergy 1 year after 4 doses

**DOI:** 10.1186/1939-4551-6-S1-P162

**Published:** 2013-04-23

**Authors:** Roderick Peter Hafner, Peter Couroux, Annemarie Salapatek, Pascal Hickey, Paul Laidler, Mark Larché, Deepen Patel

**Affiliations:** 1 Circassia Limited, Oxford, UK; 2 Cetero Research, Missisauga, ON, Canada; 3 Adiga Life Sciences Inc., Hamilton, ON, Canada; 4 Department of Medicine, Mcmaster University, Hamilton, ON, Canada

## Background

Previously, we identified a series of T-cell epitopes from the major cat allergen Fel d1 and showed these were safe and well-tolerated when administered to cat allergic subjects. In the current study, we evaluated persistence of the treatment effect (tolerance) one year after start of dosing for two treatment regimens with the same cumulative dose.

## Methods

Subjects underwent Baseline Challenge in an Environmental Exposure Chamber (EEC) on 4 consecutive days, consisting of 3-hour allergen exposures (Fel d1 50.19±3.70ng/m3). Subjects scored four nasal and four ocular symptoms every 30 minutes each on a scale of 0-3. 202 subjects were randomised to placebo, 8x3nmol Cat Peptide Antigen Desensitisation (Cat-PAD) 2weeks(wk) apart or 4x6nmol Cat-PAD 4wk apart. Subjects re-attended the EEC for 4 consecutive days, of 3-hours, 18-22wk after the start of treatment. 50-54wk after commencement of treatment subjects were invited to participate in a blinded follow-on study without further dosing. 89 subjects were enrolled and had a further 4 consecutive days, of 3-hours in the EEC.

## Results

4x6nmol Cat-PAD showed a mean change in the Total Rhinoconjunctivitis Symptom Score (TRSS) at the 50-54wk EEC visit of -6.78±5.71 versus a change of -3.89±5.56 on 8x3nmol and -2.91±5.56 on placebo. The change in TRSS score for 4x6nmol was statistically significantly different to placebo (p=0.01) and 8x3nmol (p=0.03). For the 4x6nmol regimen, the treatment effect at 50-54w trended higher than that seen at 18-22wk (4x6nmol -5.41±5.80; placebo -2.79±5.28). At the 50-54w EEC visit treatment with 4x6nmol Cat-PAD showed a mean change in Total Nasal Symptom Score of -3.44±3.05 versus a change of -1.63±2.95 on placebo (p=0.02) and a mean change in the Total Ocular Symptom Score of -3.34±3.05 versus a change of -1.28±2.92 on placebo (p=0.01).

## Conclusions

Treatment with four injections of Cat-PAD showed a substantial reduction in patients’ overall TRSS, and the ocular and nasal components of cat allergy symptom scores in the EEC model that persisted one year after the start of treatment. Four administrations of a 6nmol dose was superior to eight administrations of a 3nmol dose and placebo. The treatment effect trended higher at one year than at 18-22wk.

